# Abstract of Proceedings of the Buffalo Medical Association

**Published:** 1873-07

**Authors:** Leon F. Harvey

**Affiliations:** Secretary


ART. III.—*Abstract of the Proceedings of the Buffalo Medical Association at Meeting held May 6th,* 1873.

The President, Dr. HAUENSTEIN, in the chair.

Members present; Drs. Strong, Cronyn, Briggs, Walsh, T. M. Johnson, L. F. Harvey.

The minutes of the last meeting were read and adopted.

Dr. F. E. L. Brecht was, upon a vote being taken, declared elected a member.

The President, Dr. HAUENSTEIN, on taken the chair expressed his surprise at being elected President of the Association, not being aware of any merits that entitled him to such distinction. He said there were a large number of members who had merits and who were better qualified than himself to perform the duties of the office. But since elected, he thanked the members for the honor they conferred upon him, and that he would, to the best of his ability, perform the duties of the office, hoping for their assistance and indulgence. This Association ought to be sustained. There is enough material to make the meetings interesting. We can do each other good and be an honor to the profession. The proceedings in past days have been interesting, and we should endeavor to keep up their reputation.

Dr. A. N. BRIGGS presented the petitions of Drs. U. C. Lynde, Geo. H. Patterson, and Alphonso Dagenais for membership.

Dr. STRONG—I would like to know the practice of the gentlemen present in post-partum hemorrhage. Have they ever adopted the method common in London and Dublin, namely, that of injecting the per-chloride of iron? And what are their views as to its usefulness?

Dr. Cronyn—Have not had a case of post-partum hemorrhage since the promulgation of this remedy. I found while abroad that it met with but little favor in England or Ireland. Dr. Smith lost a patient with its use. Dr. Barnes defends it and is the father of it; uses it as a *dernier resort* in solution, or diluted full strength. Not more than six or eight in London advocate its use. Dr. Barnes upholds it with great zeal. Dr. Playfair uses it with great caution. One or two country practitioners come to the aid of Dr.

Barnes. It is used in the hospitals but very little. Young Mr. Collins speaks of it, but it has not met with favor with him. For my own part I should hesitate to use it; have passed equally potent remedies into the cavity of the uterus, but had them under more control. Should hardly venture to use it, but might. Should wipe the cavity out with saturated solution on cotton, as Dr. Playfair does in uterine catarrh.

Dr. STRONG—My colleague, Prof. Johnson, was told in Dublin by Dr. Barnes that, failing to arrest hemorrhage with cold water injections, this method had been adopted—injecting one part of iron to four parts of water.

Dr. CRONYN—A namesake of mine at the Rotunda had tried it, but did not like it.

Dr. HAUENSTEIN—In regard to post-partum hemorrhage, I would say that some women are subject to it; with them, in nearly all confinements, it occurs. There are certain signs or peculiarities in the pains that accomplish the delivery of the child, the characteristics of which if we notice them would lead us to look for hemorrhage. A labor proceeding regularly, unattended with spasmodic efforts, is seldom followed by post-partem hemorrhage. When the pains are sudden, sharp, and spasmodic, such women are more apt to have hemorrhage. In such cases, a short time before birth of child, have given a full dose of ergot, and repeated it; have also given it to those whom I knew subject to it. Half an hour before the birth in such cases I give the ergot. I think it can be prevented in this way.

Dr. STRONG—Have doubts as to the propriety of using the iron; rely on ergot.

Dr. HAUENSTEIN—I use the ergot in nervous, excitable, spasmodic, irregular contractions. As regards injections of iron I would be one of the last to use them. It is a hazardous experiment. The flow being so copious, cannot see how the solution can come in contact with the parieties.

Dr. CRONYN—The President will probably recollect in speaking of the value of ergot in one of our meetings I spoke of the improvement there was in the combination with it of quinine. Ergot induces contraction of the circular fibres almost wholly; quinine contracts the longitudinal. If the ergot acts only on the circular fibres, there may be internal hemorrhage, and ergot is valueless. The combination produces contraction of both circular and longitudinal fibres. The value of the combination is very great in post-partem hemorrhage.

Dr. Strong—Am very much interested; would like to know if the Doctor would like to commit himself to the theory that ergot will not arrest post-partum hemorrhage; have found great difference as to the effects of medicine; have almost unlimited confidence in quinine; am an advocate of it in some diseases that are not accepted. I think the Doctor has not enough faith in ergot. In a vast majority of cases it produces contraction of both sets of fibres. I cannot account for the arrest of hemorrhage unless it does contract all the fibres. How can the doctor account for ergot arresting the hemorrhage unless contraction of both take place.

Dr. Cronyn—Don't know if I have not got myself into a physiological difficulty, but think I can get out of it. We administer ergot before the expulsion of the child if post-partem hemorrhage is anticipated. The hemorrhage is not then, but after the child is expelled. In all cases of hemorrhage stimulants are used; ice and other appliances are placed on the abdomen. The hand takes the place of my quinine, and the ergot contracts beyond question the circular fibres. (Some experimental proofs were here adduced of the action of ergot on the os, etc.) There are hundreds of cases in which ergot fails and the patients die. Does anyone suppose Dr. Barnes would propose iron if ergot does not fail; too frequently it fails, when he comes in to rescue the patient with his iron.

Dr. Strong—I still do not see that the doctor answers the question that troubles. Post-partum hemorrhages are exceptional cases —not more than one in ten. We must take from them those that are controlled by common appliances—probably three-quarters of them; never have injected anything; never have lost a patient from that cause. I do not deny that ergot has power over the circular fibres, but that it is limited to them. Does the doctor concede that there is a lessened degreee of hemorrhage when ergot is used?

Dr. CRONYN—I do not deny that it influences the longitudinal but almost exclusively it controls the circular fibres.

Dr. STRONG—If the universal effect of ergot is to lessen the flow in ordinary labor, how can we account for it but that it alters the condition of the uterus by longitudinal as well as by circular contraction?

Dr. CRONYN—After the use of the ergot the uterus is irregularly contracted, and is not smooth as when not taken.

Dr. BRIGGS—Will quinine act on the uterus as soon as ergot?

Dr. CRONYN—Quinine acts very soon, as may be seen when the patient has a temperature of 110. Quinine reduces it very soon to 100. Hence its action should be as ready in parturient women.

Dr. HAUENSTEIN—I would like to add another important remark—one that is sound. When the uterus has been expanded with twins or too much liquor, contraction is naturally slow. In such cases let the placenta remain longer than the usual time. Many make mistakes in removing the placenta. There being no hemorrhage, remove it as soon as possible. I was with a physician lately when hemorrhage followed too early removal of the placenta.

Dr. CRONYN—Dr. Strong says he never lost a patient with post-partum hemorrhage; so I too can say I never lost a case. After the child is born if the uterus is flaccid, gentle kneading thereof expels the placenta, which is a foreign substance, in my opinion after the child is born. I agree with Dr. Hauenstein in regard to delay at certain times. If there be hemorrhage do not take it away quickly.

Dr. STRONG—What is the practice of physcians abroad in the use of the forceps?

Dr. CRONYN—They use them more frequently than we do, and the French even more than the Irish and English.

Prevailing Diseases—Measles, Diarrhœa, Influenza, Pneumonia, of a bronchial typhoid type, typhoid fever.

On motion, adjourned.

LEON F. HARVEY, Secretary.

